# Prevalence and related risks of cyberbullying and its effects on adolescent

**DOI:** 10.1186/s12888-023-04542-0

**Published:** 2023-01-14

**Authors:** Gassem Gohal, Ahmad Alqassim, Ebtihal Eltyeb, Ahmed Rayyani, Bassam Hakami, Abdullah Al Faqih, Abdullah Hakami, Almuhannad Qadri, Mohamed Mahfouz

**Affiliations:** 1 Pediatric Department, Faculty of Medicine, Jazan University, Jazan, Saudi Arabia; 2 Family and Community Medicine Department, Faculty of Medicine, Jazan University, Jazan, Saudi Arabia; 3 Medical Intern, Faculty of Medicine, Jazan University, Jazan, Saudi Arabia

**Keywords:** Cyberbullying, Psychological effects, Adolescents, Public health, Mental Health, Saudi Arabia

## Abstract

**Background:**

Cyberbullying is becoming common in inflicting harm on others, especially among adolescents. This study aims to assess the prevalence of cyberbullying, determine the risk factors, and assess the association between cyberbullying and the psychological status of adolescents facing this problem in the Jazan region, Saudi Arabia.

**Methods:**

A cross-sectional study was conducted on 355 students, aged between 12–18 years, through a validated online questionnaire to investigate the prevalence and risk factors of cyberbullying and assess psychological effects based on cyberbullying questionnaire and Mental Health Inventory-5 (MHI-5) questions.

**Results:**

The participants in this study numbered 355; 68% of participants were females compared to 32% were males. Approximately 20% of the participants spend more than 12 h daily on the Internet, and the estimated overall prevalence of cyberbullying was 42.8%, with the male prevalence slightly higher than females. In addition, 26.3% of the participants were significantly affected in their academic Performance due to cyberbullying. Approximately 20% of all participants considered leaving their schools, 19.7% considered ceasing their Internet use, and 21.1% considered harming themselves due to the consequences of cyberbullying. There are essential links between the frequency of harassment, the effect on academic Performance, and being a cyber victim.

**Conclusions:**

Cyberbullying showed a high prevalence among adolescents in the Jazan region with significant associated psychological effects. There is an urgency for collaboration between the authorities and the community to protect adolescents from this harmful occurrence.

## Introduction

Cyberbullying is an intentional, repeated act of harm toward others through electronic tools; however, there is no consensus to define it [1–3]. With the surge in information and data sharing in the emerging digital world, a new era of socialization through digital tools, and the popularization of social media, cyberbullying has become more frequent than ever and occurs when there is inadequate adult supervision [4, 5]. A large study that looked at the incidence of cyberbullying among adolescents in England found a prevalence of 17.9%, while one study conducted in Saudi Arabia found a prevalence of 20.97% [6, 7]. Cyberbullying can take many forms, including sending angry, rude, or offensive messages; intimidating, cruel, and possibly false information about a person to others; sharing sensitive or private information (outing); and exclusion, which involves purposefully leaving someone out of an online group [8]. Cyberbullying is influenced by age, sex, parent–child relationships, and time spent on the Internet [9, 10]. Although some studies have found that cyberbullying continues to increase in late adolescence, others found that cyberbullying tends to peak at 14 and 15 years old before decreasing through the remaining years of adolescence [11–13].

The COVID-19 epidemic has impacted the prevalence of cyberbullying since social isolation regulations have reduced face-to-face interaction, leading to a significant rise in the use of social networking sites and online activity. As a result, there was a higher chance of experiencing cyberbullying [14].

Unlike traditional Bullying, which usually only occurs in school and is mitigated at home, victims of cyberbullying can be contacted anytime and anywhere. Parents and teachers are seen as saviors in cases of traditional Bullying. Simultaneously, in cyberbullying, children tend to be reluctant to tell adults for fear of losing access to their phones and computers, so they usually hide the cyberbullying incident [15]. Reports show that cyberbullying is a form of harm not easily avoided by the victim. In addition, in the cyber form of Bullying, identification of the victim and the perpetrator is generally challenging compared to traditional Bullying; this makes an accurate estimation of the problem widely contested [16, 17].

There is growing evidence that is cyberbullying causes more significant levels of depression, anxiety, and loneliness than traditional forms of Bullying. A meta-analysis examining the association between peer victimization, cyberbullying, and suicide in children and adolescents indicates that cyberbullying is more intensely related to suicidal ideation than traditional Bullying [18]. Moreover, the significant problem is that cyberbullying impacts adolescent due to its persistence and recurrence. A recent report in Saudi Arabia indicated a growing rise in cyberbullying in secondary schools and higher education, from 18% to approximately 27% [19]. In primary schools and kindergartens in Saudi Arabia, we were not surprised to find evidence that children were unaware that cyberbullying is illegal. Although the study showed an adequate awareness of the problem in our country, Saudi Arabia, there were relatively significant misconceptions [20].

Adolescents' emotional responses to cyberbullying vary in severity and quality. However, anger, sadness, concern, anxiety, fear, and depression are most common among adolescent cyber victims [21]. Moreover, cyberbullying may limit students' academic Performance and cause higher absenteeism rates [22]. Consequently, this study aims to assess the prevalence of cyberbullying, determine the risk factors, and establish the association between cyberbullying and the psychological status of adolescents. We believe our study will be an extension of and significantly add to the literature regarding the nature and extent of cyberbullying in the Jazan region of Saudi Arabia.

## Methods

A descriptive cross-sectional study was carried out in the Jazan region, a province of the Kingdom of Saudi Arabia. It is located on the tropical Red Sea coast of southwestern Saudi Arabia.

### Design and participants

A descriptive cross-sectional study was carried out in the Jazan region, a province of the Kingdom of Saudi Arabia. It is located on the tropical Red Sea coast of southwestern Saudi Arabia. The study targeted adolescents (12–18 years old) who use the Internet to communicate in the Jazan region. The main inclusion criteria are adolescents between 12–18 years who use the Internet and agree to participate; however, it excludes adolescents not matching the inclusion criteria or those refusing to participate in the study. If participants were under 16, the parent and/or legal guardian should be notified. A sample of participants was estimated for this study, and the ideal sample size was calculated to be 385 using the Cochran formula, *n* = (z) ^2^ p (1 – p) / d^2^. Where: p = prevalence of cyberbullying 50%, z = a 95% confidence interval, d = error of not more than 5%. A convenience sample was used to recruit the study participants. A self-administrated online questionnaire was used to collect the study information from May to December 2021.

The ethical approval for this study was obtained from The Institute Review Board (IRB) of Jazan University (Letter v.1 2019 dated 08/04/2021). Informed consent was acquired from all participants and was attached to the beginning of the form and mandatory to be read and checked before the participant proceeded to the first part of the questionnaire. For the participants under 16, informed consent was obtained from a parent and legal guardian.

### Procedure of data collection and study measures

An Arabic self-administrated online questionnaire was used for this research. This anonymous online survey instrument was based on (Google Forms). The study team distributed the questionnaire to the participants through school teachers. The research team prepared the study questionnaire and chose the relevant cyberbullying scale questions from similar studies [5, 6]. The questionnaire was translated by two bilingual professionals to ensure the accuracy and appropriateness of the instrument wording. A panel of experts then discussed and assessed the validity and suitability of the instrument for use on adolescents. The panel also added and edited a few questions to accommodate the local culture of Saudi students. It was validated with a pilot study that included 20 participants. The questionnaire was divided into three main sections. The first part of the questionnaire contains the basic participant information, including gender, age, nationality, school grade, residence, and information about family members and the mother's occupation and education. The mother's level of education was considered as it found that mothers' low levels of education specifically had a detrimental impact on the cyberbullying process [23]. The second section explores the participant's definition of cyberbullying, questions regarding exposure to cyberbullying as a victim or by bullying another person, and questions considering the possible risk factors behind cyberbullying. The last section explores how cyberbullying affects adolescents psychologically based on the standardized questionnaire Mental Health Inventory-5 (MHI-5). MHI-5 is a well-known, valid, reliable, and brief international instrument for assessing mental health in children and adolescents (such as satisfaction, interest in, and enjoyment of life) and negative aspects (such as anxiety and depression) [24]. It is composed of five questions, as shown in Table 1. There are six options available for each question, ranging from "all the time" (1 point) to "none of the time" (6 points); therefore, the adolescent's score varies between five and 30. These questions assess both negative and positive qualities of mental health, as well as questions about anxiety and depression. By adding all the item scores and converting this score to a scale ranging from 0 to 100, the final MHI-5 score is determined, with lower scores indicating more severe depressive symptoms. The value for which the sum of sensitivity and specificity was utilized to establish the ideal cut-off score for MHI-5 in many similar studies was reviewed to reach an optimal conclusion. Therefore, we considered all cut-off values with associated sensitivities and specificities of various MHI-5 cut-off points previously employed among adolescents in similar studies and compared them to conclude that MHI-5 = 70 as our cut points. So the presence of depressive symptoms is considered with an MHI-5 cut-off score of ≤ 70 [25].

**Table 1:** Factor Analysis of the Arabic Version of the Mental Health Inventory – 5 (MHI-5) (*n* = 355)

Questions		Factorial Loading	Total Variance Explained
1	During the past month, how much of the time were you a happy person?	-.735	Eigenvalue = 2.838 Explained Variance = 56.766
2	How much of the time, during the past month, have you felt calm and peaceful?	-.720	
3	How much of the time, during the past month, have you been a very nervous person?	.688	
4	How much of the time, during the past month, have you felt downhearted and depressed?	.824	
5	How much of the time, during the past month, have you felt depressed that nothing could cheer you up?	.792	

Kaiser–Meyer–Olkin Measure of Sampling Adequacy = 0.700

Bartlett’s Test of Sphericity, Chi-Square = 739.84 *p* < 0.001

The Questionnaires were initially prepared in English and then translated into Arabic. A native speaker with fluency in English (with experience in translation) converted the questionnaire from the initial English version into Arabic. Then, we performed a pilot study among 20 participants to ensure the readability and understandability of the questionnaire questions. We also assessed the internal consistency of the questionnaire based on Cronbach’s alpha, which produced an acceptable value of 0.672. The internal consistency for Mental Health Inventory-5 (MHI-5) was reported at 0.557. In order to assess the factor structure of the Arabic-translated version of the (MHI-5) questionnaire, a factor analysis was conducted. The factor loading of the instrument is shown in Table 1. Using principal component analysis and the varimax rotation method, we found a one-component solution explaining 56.766% of the total variance. All items loaded on the first factor ranged from (0.688 to 0.824), which confirms that a single factor has explained all the items of the scale. In addition, Bartlett’s test of sphericity was found significant (*p* < 0.001).

### Data presentation & statistical analysis

Simple tabulation frequencies were used to give a general overview of the data. The prevalence of cyberbullying was presented using 95% C.I.s, and the Chi-squared test was performed to determine the associations between individual categorical variables and Mental Health. The univariate and multivariate logistic regression model was derived, and unadjusted and adjusted odds ratios (OR) and their 95% confidence intervals (C.I.s) were calculated. A *P*-value of 0.05 or less was used as the cut-off level for statistical significance. The statistical analysis was completed using SPSS ver. 25.0 (SPSS Inc. Chicago, IL, USA) software.

## Results

The distributed survey targeted approximately 385 students, but the precise number of respondents to the questionnaire was 355 (92% response rate), with 68% of female students responding, compared to 32% of male students. More than half of the respondents were secondary school students, with a nearly equal mix of respondents living in cities and rural areas. Table 2 demonstrates that 20% of the participants spend more than 12 h daily on the Internet and electronic gadgets, while only 13% spend less than two hours.

**Table 2:** Socio-demographic characteristics of participants

Characteristics		*N* = 355			
		N	%	Mean	SD
Gender	Male	113	32%	63.22	(22.10)
	Female	242	68%	58.86	(25.24)
Age groups	12–13 years	126	35%	66.63	(23.91)
	14–15 years	105	30%	61.68	(23.70)
	16-–18 years	124	35%	52.55	(23.36)
Grades	Sixth Primary	26	7%	74.46	(17.53)
	1st Intermediate	29	8%	62.21	(27.31)
	2nd Intermediate	45	13%	61.33	(25.78)
	3rd Intermediate	57	16%	62.39	(23.30)
	1st Secondary	30	9%	64.00	(21.68)
	2nd Secondary	81	23%	56.54	(26.08)
	3rd Secondary	84	24%	55.05	(22.56)
Mode of living	Town	173	49%	58.45	(23.96)
	Village	182	51%	61.96	(24.63)
Mother’s level of education	Illiterate	67	19%	55.34	(25.53)
	Primary	55	15%	58.40	(24.99)
	Intermediate	34	10%	59.29	(23.10)
	Secondary	70	20%	65.66	(23.25)
	University	129	36%	60.90	(24.06)
Mother’s occupation	HW	267	75%	60.93	(24.27)
	Retired	10	3%	69.20	(15.78)
	Government employee	68	19%	55.65	(25.60)
	Private sector	3	1%	70.67	(16.65)
	Own business	7	2%	61.71	(24.42)
Father’s occupation	Not working	45	13%	54.93	(28.79)
	Retired	100	28%	60.08	(24.43)
	Government employee	160	45%	59.48	(22.75)
	Private sector employee	24	7%	69.50	(24.25)
	Own business	26	7%	66.31	(23.60)
Hours spent on electronic devices	Less than 2 h	47	13%	59.74	(25.94)
	2–4 h	93	26%	64.90	(24.61)
	4–8 h	84	24%	61.24	(22.05)
	8–12 h	61	17%	60.07	(21.50)
	More than 12 h	70	20%	53.37	(26.78)

Abbreviations: SD = standard deviation

As demonstrated in Table 3, the total prevalence of cyberbullying was estimated to be 42.8%, with male prevalence somewhat higher than female prevalence. Additional variables, such as the number of hours spent on the Internet, did not affect the prevalence. Table 4 shows the pattern and experience of being cyberbullied across mental health levels, as measured by the MHI-5.

**Table 3:** Prevalence of cyberbullying among adolescents in the Jazan region

Characteristics		Adolescents Being Cyberbullied		95% C.I		*p*-value
		***n***	**%**	**Lower**	**Upper**	
Gender	Male	54	(47.8)	38.7%	57.0%	0.096
	Female	98	(40.5)	34.5%	46.8%	
Age Groups	12–13 years	45	(35.7)	27.7%	44.3%	0.053
	14–15 years	44	(41.9)	32.8%	51.5%	
	16–18 years	63	(50.8)	42.1%	59.5%	
Grades	Sixth Primary	9	(34.6)	18.7%	53.7%	0.452
	1st Intermediate	10	(34.5)	19.3%	52.6%	
	2nd Intermediate	18	(40.0)	26.7%	54.6%	
	3rd Intermediate	26	(45.6)	33.2%	58.5%	
	1st Secondary	9	(30.0)	16.0%	47.7%	
	2nd Secondary	40	(49.4)	38.7%	60.1%	
	3rd Secondary	39	(46.4)	36.0%	57.1%	
Mode of living	Town	70	(40.5)	33.4%	47.9%	0.382
	Village	82	(45.1)	38.0%	52.3%	
Mother’s level of education	Illiterate	32	(47.8)	36.1%	59.6%	0.231
	Primary	17	(30.9)	19.9%	43.9%	
	Intermediate	18	(52.9)	36.5%	68.9%	
	Secondary	28	(40.0)	29.1%	51.7%	
	University	57	(44.2)	35.8%	52.8%	
Mother’s occupation	HW	106	(39.7)	34.0%	45.7%	0.184
	Retired	4	(40.0)	15.3%	69.6%	
	Government employee	35	(51.5)	39.7%	63.1%	
	Private sector	2	(66.7)	17.7%	96.1%	
	Own business	5	(71.4)	35.2%	93.5%	
Father’s occupation	Not working	23	(51.1)	36.8%	65.3%	0.424
	Retired	44	(44.0)	34.6%	53.8%	
	Government employee	69	(43.1)	35.6%	50.9%	
	Private sector employee	7	(29.2)	14.1%	48.9%	
	Own business	9	(34.6)	18.7%	53.7%	
Hours spent on electronic devices	Less than 2 h	17	(36.2)	23.6%	50.4%	0.897
	2–4 h	39	(41.9)	32.3%	52.1%	
	4–8 h	38	(45.2)	34.9%	55.9%	
	8–12 h	27	(44.3)	32.3%	56.8%	
	More than 12 h	31	(44.3)	33.1%	56.0%	
**Overall Prevalence**		**152**	**(42.8)**	**37.7%**	**48.0%**	

^#^ *p*-value is based on Pearson's Chi-square test, Abbreviations: CI = confidence interval

**Table 4:** Pattern and experience of being cyberbullied among adolescents according to a mental health level based on MHI-5

Characteristics		All		Mental Health				p-value
				Low MHI-5 ≤ 70		High MHI-5 > 70		
		N	%	N	%	N	%	
Hours spent on electronic devices	Less than 2 h	17	(11.2)	10	(58.8)	7	(41.2)	0.307
	2–4 h	39	(25.7)	21	(53.8)	18	(46.2)	
	4–8 h	38	(25.0)	25	(65.8)	13	(34.2)	
	8–12 h	27	(17.8)	21	(77.8)	6	(22.2)	
	More than 12 h	31	(20.4)	24	(77.4)	7	22.6%	
Have you been cyber bullied before?	Yes	152	(42.8)	101	(66.4)	51	(33.6)	< 0.001
	No	203	(57.2)	84	(41.4)	119	(58.6)	
Is the offender known to you or unknown?	Unknown person	70	(47.6)	43	(61.4)	27	(38.6)	0.127
	Known person	77	(52.4%)	55	(71.4)	22	(28.6)	
Frequency of harassment?	Days	83	(38.8)	48	(57.8)	35	(42.2)	0.046
	Weeks	11	(5.1)	5	(45.5)	6	(54.5)	
	Months	23	(10.7)	13	(56.5)	10	(43.5)	
	Years	97	(45.3)	72	(74.2)	25	(25.8)	
Has your academic Performance been affected because of being cyberbullied?	Yes	40	(26.3)	36	(90.0)	4	(10.0)	< 0.001
	No	112	(73.7)	65	(58.0)	47	(42.0)	
Have you considered leaving school because of being cyberbullied?	Yes	30	(19.7)	25	(83.3)	5	(16.7)	0.025
	No	122	(80.3)	76	(62.3)	46	(37.7)	
Have you considered blocking the bullying person?	Yes	69	(45.4)	54	(78.3)	15	(21.7)	0.005
	No	83	(54.6)	47	(56.6)	36	(43.4)	
Have you considered stopping the use of electronic devices?	Yes	30	(19.7)	25	(83.3)	5	(16.7)	0.029
	No	122	(80.3)	76	(62.3)	46	(37.7)	
Have you considered harming yourself because of being cyberbullied?	Yes	32	(21.1)	29	(90.6)	3	(9.4)	0.001
	No	120	(78.9)	72	(60.0)	48	(40.0)	
Have you told anyone about your cyberbullying experience?	Yes	67	(44.7)	44	(65.7)	23	(34.3)	0.697
	No	83	(55.3)	57	(68.7)	26	(31.3)	
Have you ever asked for help?	Yes	87	(57.2)	54	(62.1)	33	(37.9)	0.186
	No	65	(42.8)	47	(72.3)	18	(27.7)	
Have you ever committed Cyberbullying?	No	314	(88.5)	160	(51.0)	154	(49.0)	0.227
	Yes	41	(11.5)	25	(61.0)	16	(39.0)	

^#^ *p*-value is based on Pearson's Chi-square test, Abbreviations: MHI-5 = the Mental Health Inventory-5

Academic Performance was significantly affected due to cyberbullying in 26.3% of the participants. Furthermore, approximately 20% of all participants considered leaving their schools for this reason. Moreover, 19.7% of the participants thought of stopping using the Internet and electronic devices, while 21.1% considered harming themselves due to the effects of cyberbullying. Regarding associations between various variables and psychological effects using the MHI-5, there are significant associations between whether the participant has been a cyber victim before (cOR 2.8), the frequency of harassment (cOR 1.9), academic Performance (cOR 6.5), and considering leaving school as a result of being a cyber victim (cOR 3.0). In addition, by using univariate logistic regression analysis, there are significant associations between the psychological effects and the participant's thoughts of getting rid of a bully (cOR 2.8), thinking to stop using electronic devices (cOR 3.0), and considering hurting themselves as the result of cyberbullying (cOR 6.4). In addition, the use of the multivariate logistic regression analysis showed that frequency of harassment was the only statistically significant predictor of mental health among adolescents (aOR 2.8). Other variables continue to have higher (aORs) but without statistical significance. All these results are demonstrated in Table 4.

## Discussion

Cyberbullying prevalence rates among adolescents vary widely worldwide, ranging from 10% to more than 70% in many studies. This variation results from certain factors, specifically gender involvement, as a decisive influencing factor [26, 27]. Our study found a prevalence of 42.8% (95% confidence interval (CI): 37.7–48), which is higher than the median reported prevalence of cyberbullying of 23.0% in a scoping review that included 36 studies conducted in the United States in adolescents aged 12 to 18 years old [28]. A systematic review found that cyberbullying ranged from 6.5% to 35.4% [3]. These two studies gathered data before the COVID-19 pandemic. When compared to recent studies, it was found that cyberbullying increased dramatically during the COVID-19 era [29, 30]. Subsequently, with the massive mandate of world online communication in teaching and learning, young adolescents faced a large amount of cyberspace exposure with all risk-related inquiries. Psychological distress due to COVID-19 and spending far more time on the Internet are vital factors in this problem, which might be a reasonable explanation for our results.

There is insufficient data to compare our findings to the Arab world context, notably Saudi Arabia. Although, according to one study done among Saudi Arabian university students, the prevalence was 17.6%. [31]. we discovered a considerable discrepancy between this prevalence and our findings, and the decisive explanation is the difference in the target age group studied. Age is a crucial risk factor for cyberbullying, and according to one study, cyberbullying peaks at around 14 and 15 years of age and then declines in late adolescence. Thus, a U-inverted relation exists between prevalence and age [11–13, 32].

In our study, males reported being more vulnerable to cyberbullying despite there being more female participants; this inconsistent finding with previous literature requires further investigation. A strong, but not recent, meta-analysis in 2014 reported that, in general, males are likely to cyberbully more than females. Females were more likely to report cyberbullying during early to mid-adolescence than males [11]. This finding presents a concern for males reporting lower than females’ results in our data and raises some questions about whether cultural or religious conservative values play a role.

Increased Internet hours are another risk factor in this study and were significantly associated with cyberbullying. Specifically, it was likely to be with heavy Internet users (> 12 h/day); a similar result was well documented in one equivalent study [3]. Notably, while some studies have reported that those living in city areas are more likely to be cyberbullying victims than their counterparts from suburban areas [3], our observations reported no significant influence of this factor on the prevalence of cyberbullying.

According to a population-based study on cyberbullying and teenage well-being in England, which included 110,000 pupils, traditional Bullying accounted for more significant variability in mental well-being than cyberbullying. It did, however, conclude that both types of Bullying carry a risk of affecting mental health [33]. We confirmed in this study that multiple occurrences of cyberbullying and the potential for being a victim are risk factors influencing mental health (*P* < 0.001). Moreover, the frequency of harassment also shows a significant, influential effect. The victim's desire to be free from the perpetrator carrying out the cyberbullying is probably an alarming sign and a precursor factor for suicidal ideation; we reported that nearly half of the participants wished they could get rid of the perpetrators. Furthermore, more than 20% of participants considered harming themselves due to cyberbullying; this result is consistent with many studies that linked cyberbullying and self-harm and suicidal thoughts [34–36].

Adolescence is a particularly vulnerable age for the effects of cyberbullying on mental health. In one Saudi Arabian study, parents felt that cyberbullying is more detrimental than Bullying in the schoolyard and more harmful to their children's mental health. According to them, video games were the most popular social platform for cyberbullying [37]. Both cross-sectional and longitudinal research shows a significant link between cyberbullying and emotional symptoms, including anxiety and depression [38, 39]. Therefore, we employed the MHI-5 to measure the mental impact of cyberbullying on adolescents in this study. Overall, the MHI-5 questionnaire showed relatively high sensitivity in detecting anxiety and depression disorders for general health and quality of life assessments. The questions listed happy times, peacefulness, and sensations of calmness, in addition to episodes of anxiousness, downheartedness, and feelings of depression, as given in Table 1.

Cyberbullying has been well-documented to affect the academic achievement of the victim adolescents. Therefore, bullied adolescents are likelier to miss school, have higher absence rates, dislike school, and report receiving lower grades. According to one meta-analysis, peer victimization has a significant negative link with academic achievement, as measured by grades, student performance, or instructor ratings of academic achievement [40]. In our investigation, we reported that up to 20% of participants considered leaving their schools due to the adverse effects of cyberbullying (cOR 3.0) and wished they could stop using the Internet; 26% of participants felt that their school performance was affected due to being cyber victims (cOR 6.5). The results of the univariate analysis showed a high odd ratio related to school performance and a willingness to leave school. This conclusion indicates the likelihood of these impacts specifically with a significant p-value, as shown in Table 5.

**Table 5:** Uni-variate and multivariate logistic regression analyses showing associations between various variables of adolescent cyberbullying and mental health level

Characteristics		Uni-variate				Multivariate			
		cOR	95% C.I		*p*-value	aOR	95% C.I		*p*-value
			Lower	Upper			Lower	Upper	
Age (years)		1.2	1.12	1.4	< 0.001	1.2	0.9	1.5	0.183
Gender	Female*	1				1			
	Male	1.9	0.9	3.7	0.081	2.2	0.9	5.2	0.075
Being Cyberbullied Before	No*								
	Yes	2.8	1.8	4.3	< 0.001	-	-	-	-
Academic Performance affected Cyberbullying	No*	1							
	Yes	6.5	2.2	19.5	0.001	2.5	0.7	9.4	0.174
Frequency of harassments	Days or Weeks*	1				1			
	Months or years	1.9	1.1	3.3	0.029	2.8	1.2	6.4	0.017
Thinking about leaving school as a result of being Cyberbullied	No*	1							
	Yes	3.0	1.1	8.5	0.035	-	-	-	-
Have you thought about getting rid of the bully on you?	No*	1							
	Yes	2.8	1.3	5.7	0.006	1.9	0.8	4.4	0.128
Thinking to stop using Electronic Devices	No*	1				1			
	Yes	3.0	1.1	8.5	0.035	1.3	0.3	4.8	0.737
Thinking about hurting yourself as a result of being Cyberbullied	No*	1							
	Yes	6.4	1.9	22.3	0.003	3.6	0.9	15.0	0.078

^*^ Reference category, *CI* Confidence Interval, *cOR* Crude Odds ratio, *aOR* Adjusted Odds ratio

In this study, approximately 88% of the participants were cyber victims compared to only 11% of cyberbullying perpetrators who committed this act on their peers. Mental health affection is well-reported in many studies on cyber victims with higher depression rates than cyberbullying perpetrators [41, 42]. However, other studies indicate that cyberbullying victims are not the only ones affected; harm is also extended to involve perpetrators. Cyberbullying perpetrators have high-stress levels, poor school performance, and an increased risk of depression and alcohol misuse. Furthermore, research shows that adolescents who were victims or perpetrators of cyberbullying in their adolescence continue to engage in similar behavior into early adulthood [43, 44].

### Limitations of the study

Although the current study found a high prevalence and positive connections among variables, it should be emphasized that it was conducted on a determinate sample of respondents, 11 to 18 years old. Therefore, the results could not be generalized for other samples, age groups, and communities from other cultures and contexts. In addition, it was limited to adolescent survey responses, did not include parents' and caretakers' viewpoints, and failed to include other risk factors such as divorce and financial status. We believe future studies should consider parents' perspectives and more analysis of perpetrators' characteristics. Moreover, self-reported tools are susceptible to social desirability bias, which can influence test item responses. As a result, future research should employ a variety of monitoring and evaluation metrics and larger potential populations and age ranges. Another limitation of this analysis is that we cannot make conclusive inferences regarding gender and exact prevalence because male adolescents had a lower response rate than female adolescents, suggesting that males might be more sensitive to disclosing these issues.

## Conclusion

Even though experts in the social sciences typically research cyberbullying, it is crucial to investigate it from a clinical perspective because it significantly affects mental health. Adolescents' lives have grown increasingly centered on online communication, which provides several possibilities for psychological outcomes and aggressive actions such as cyberbullying. Stress, anxiety, depressive symptoms, suicidal ideation, and deterioration in school performance are all linked to cyberbullying. Therefore, we emphasize the need for parents and educators to be conscious of these dangers and be the first line of protection for the adolescent by recognizing, addressing, and solving this problem. Furthermore, we urge the responsibility of pediatricians, physicians, and psychiatric consultants to create a comfortable atmosphere for adolescents to disclose and report this problem early and raise awareness of the problem in their communities. Furthermore, practical strategies for dealing with such occurrences involving health, education, and law authorities, should be supported to tackle this problem, which can affect the adolescent mentally and academically. Lastly, to decide how to intervene most effectively, more research must be done on the many methods to assess how schools, communities, and healthcare providers tackle cyberbullying.

## Data Availability

The authors ensure that the data supporting the results of this study are available within the article. The raw data for the study will be obtainable from the corresponding author upon reasonable demand.
